# Responsive Expression of MafF to *β*-Amyloid-Induced Oxidative Stress

**DOI:** 10.1155/2020/8861358

**Published:** 2020-12-07

**Authors:** Xiaoxuan Wang, Yu Zhang, Xinkun Wan, Chenjia Guo, Jing Cui, Jing Sun, Liang Li

**Affiliations:** Department of Pathology, School of Basic Medical Sciences, Capital Medical University, Beijing 100069, China

## Abstract

The small musculoaponeurotic fibrosarcoma (sMaf) proteins MafF, MafG, and MafK are basic region leucine zipper- (bZIP-) type transcription factors and display tissue- or stimulus-specific expression patterns. As the oxidative stress reactive proteins, sMafs are implicated in various neurological disorders. In the present study, the expressions of sMafs were investigated across five databases gathering transcriptomic data from 74 Alzheimer's disease (AD) patients and 66 controls in the Gene Expression Omnibus (GEO) database. The expression of MafF was increased in the hippocampus of AD patients, which was negatively correlated with the expression of the glutamate cysteine ligase catalytic subunit (GCLC). Furthermore, MafF was significantly increased in patients with Braak stage V-VI, compared to those with Braak stage III-IV. *β*-Amyloid (A*β*), a strong inducer of oxidative stress, plays a crucial role in the pathogenesis of AD. The responsive expressions of sMafs to A*β*-induced oxidative stress were studied in the APP/PS1 mouse model of AD, A*β* intrahippocampal injection rats, and several human cell lines from different tissue origins. This study revealed that only the induction of MafF was accompanied with reduction of GCLC and glutathione (GSH). MafF knockdown suppressed the increase of GSH induced by A*β*. Among sMafs, MafF is the most responsive to A*β*-induced oxidative stress and might potentiate the inhibition of antioxidation. These results provide a better understanding of sMaf modulation in AD and highlight MafF as a potential therapeutic target in AD.

## 1. Introduction

The musculoaponeurotic fibrosarcoma (Maf) proteins belong to the family of the basic region leucine zipper- (bZIP-) type transcription factors, also including activator protein- (AP-) 1 factors, CREB/ATF, CNC, C/EBP, and PAR [1, 2]. There are two groups in the Maf family. The large Maf proteins, including v-Maf, c-Maf, MafB, and Nrl, contain a distinctive acidic domain suggestive of transcriptional activation properties. The small Maf (sMaf) proteins, including MafF, MafG, and MafK, present high degrees of homology. They are predominantly localized in the nucleus and have emerged as important transcription regulators, although they lack activation domains [3, 4]. Nuclear factor (erythroid-derived 2)-like 2 (Nrf2) belongs to the bZIP family and is a master regulator of antioxidant and xenobiotic metabolizing enzymes [5–7]. Because Nrf2 cannot bind to the DNA, sMafs are indispensable partners that are required by Nrf2 to exert its functions. In response to oxidative and electrophilic stresses, Nrf2 translocates to the nucleus and heterodimerizes with sMafs to activate specific target genes such as glutamate cysteine ligase catalytic subunit (GCLC). This activation promotes the production of antioxidants such as glutathione (GSH). sMafs can also form homodimers that act as transcriptional repressors [8, 9]. One study suggested that all three sMaf genes play important roles in oxidative stress. Yet, even though the three genes can be coexpressed in the same cell line, their expressions seem to be regulated by distinct mechanisms [10].

Many studies reported the relevance of sMafs in a broad range of human pathologies, such as neurological disorders [11], chronic myeloid leukemia [12], diabetes [13], lung cancers [14], and hepatocellular carcinoma [15]. Although the causes of neurodegenerative diseases remain unclear, growing evidences point toward oxidative injury as an important pathogenic mechanism. Alzheimer's disease (AD) manifests by cognitive dysfunction and memory impairment. The pathological characteristics of AD are the formations of extracellular *β*-amyloid (A*β*) plaques and intraneuronal deposits of neurofibrillary tangles (NFTs). A*β* is produced by cleavage of the transmembrane glycoprotein amyloid precursor protein (APP) through the sequential actions of *β*-secretases and presenilin-dependent *γ*-secretases [16]. A*β* is a strong oxidative stress inducer that promotes the formation of free radicals and causes damages to nerve cells [17]. Extensive data demonstrate that A*β* overproduction in the brains of AD patients and APP/PS1 mice is positively correlated with the level of oxidative stress [18] and that the Nrf2-sMaf signal pathway is severely inhibited in late AD patients [19–21]. Despite the potential link between oxidative stress, sMafs, and AD, the functions of sMafs in the brain, especially in AD conditions, are rarely documented.

In the present study, we compared the transcriptomes of 74 AD patients and 66 controls taken from five databases. Since A*β* is a main cause for the various pathological changes observed in AD, we investigated the expression levels of MafF, MafG, and MafK in APP/PS1 transgenic mice, A*β* intrahippocampal injection rat model, and A*β*-treated several human cell lines from different tissue.

## 2. Materials and Methods

### 2.1. Data Collection

Transcriptomic data from 74 AD patients and 66 controls were collected from the databases GSE29378 (https://www.ncbi.nlm.nih.gov/geo/query/acc.cgi?acc=GSE29378), GSE36980 (https://www.ncbi.nlm.nih.gov/geo/query/acc.cgi?acc=GSE36980), GSE28146 (https://www.ncbi.nlm.nih.gov/geo/query/acc.cgi?acc=GSE28146), GSE48350 (https://www.ncbi.nlm.nih.gov/geo/query/acc.cgi?acc=GSE48350), and GSE5281 (https://www.ncbi.nlm.nih.gov/geo/query/acc.cgi?acc=GSE5281). The cross-platform normalized data were downloaded from the AlzData database [22–27]. The basal clinical information related to the samples was provided in Supplement Table S1.

### 2.2. Animals

Nine-month-old male APPswe/PS1dE9 (APP/PS1) mice (Ethical approval AEEI-2019-081, Beijing HFK Bioscience Co., Ltd. Beijing, China) and 12-week-old adult male Sprague-Dawley (SD) rats (Ethical approval AEET-2017-103, Beijing Vital River Experimental Animal Technology Co., Ltd, Beijing, China) were used in this study. All animals were housed in the Experimental Animal Center of the Capital Medical University (Beijing, China) under standard laboratory conditions (22°C–24°C, 40%–60% relative humidity) with regular 12 h light/dark cycles. All experimental procedures were performed in compliance with the Guidance for the Care and Use of Laboratory Animals formulated by the Ministry of Science and Technology of China.

### 2.3. Cell Culture and Treatment

SH-SY5Y neuroblastoma, HepG2 liver hepatocellular carcinoma, and A549 alveolar basal epithelial human cell lines were cultured in Dulbecco minimum essential medium (DMEM) (Gibco, USA) containing 10% fetal bovine serum (Gibco) at 37°C in incubator supplied with 5% CO_2_. Both A*β*_1-42_ peptides (China Peptides Co., Ltd.) and scrambled A*β* peptides with the same amino acid composition as A*β*_1-42_ but in randomized sequences were aggregated before use by incubation in dimethyl sulfoxide (DMSO) at 37°C for 72 h [28]. The oligomerization of A*β* was verified by electronic microscopy. A*β*_1-42_ or scrambled A*β* peptides were added to the medium and incubated for 48 h. Hydrogen peroxide (H_2_O_2_, Beijing Shiji, Beijing, China) or phosphate buffer saline (PBS) was added to the medium and incubated for 24 h.

### 2.4. Stereotaxic Injection of A*β*

Rats anesthetized with 2%–3% isoflurane with an animal anesthesia ventilator system (RWD Life Science Co. Ltd., Shenzhen, China) were placed onto a stereotaxic frame (RWD Life Science Co. Ltd., Shenzhen, China). A microsyringe was implanted stereotactically to the hippocampus (4.3 mm posterior to the bregma; 3.5 mm lateral from midline; and 3.3 mm ventral to bregma). A volume of 3 *μ*l containing 20 *μ*g of A*β*_1-42_ or scrambled A*β* was delivered with a stepper-motorized 10 *μ*l microsyringe at a rate of 1 *μ*l/min [28].

### 2.5. Quantitative Real-Time PCR (q-PCR)

Total RNAs were extracted from tissues and cells using RNAsimple Total RNA kit (Tiangen, Beijing, China). Reverse transcriptions were performed using FastQuant kit with gDNase (Tiangen). The q-PCR reactions were performed as previously described [28]. Each q-PCR assay was run in triplicate in a CFX96 Touch system (Bio-Rad, Hercules, CA, USA). Data were analyzed using the 2^-*ΔΔ*Ct^ method with glyceraldehyde-3-phosphate dehydrogenase (GAPDH) or beta-actin (*β*-actin) as controls. The primer sequences are listed in Table 1.

### 2.6. Western Blot

Cells or hippocampal tissues were harvested and homogenized in RIPA buffer containing a cocktail of protease inhibitors (MedChemExpress, NJ, USA). Whole-cell lysates were prepared for western blot analysis as described before [29]. The primary antibodies were as follows: rabbit anti-GCLC antibody (1 : 10000, Abcam, UK), rabbit anti-MafF antibody (1 : 1000, Proteintech Group, Rosemont, IL, USA), rabbit anti-MafG antibody (1 : 2000, Novus Biologicals, CO, USA), rabbit anti-MafK antibody (1 : 5000, Abcam), and mouse GAPDH monoclonal antibody (1 : 5000, Proteintech Group). Signals were quantified with Alpha FluorChem FC3 system (Protein Simple, San Francisco, CA, USA) and analyzed with ImageJ 16.0 (NIH, Bethesda, MD, USA).

### 2.7. Determination of GSH

Quantitative analysis of GSH levels was performed with Reduced GSH Assay kit (Nanjing Jiancheng Bioengineering Institute, Nanjing, China) according to the manufacturer's instructions. The absorbance was measured using a microplate reader (Thermo Scientific, Waltham, MA, USA) at a wavelength of 405 nm.

### 2.8. Cell Transfection

MafF siRNA was purchased from Hanbio (Shanghai, China). SH-SY5Y cells were seeded in 6-well plates. Two milliliters of transfection solution containing RNAFit and 10 nM of MafF siRNA or scrambled siRNA used as a negative control (NC) were added to the cultures. Cells were collected 48 h after transfection.

### 2.9. Statistical Analysis

Statistical analysis was carried out using the unpaired two-tailed Student *t*-test or variance analysis (ANOVA) followed by a Tukey's post hoc test using GraphPad Prism 6 software (GraphPad Software, San Diego, CA, USA) [28]. Pearson correlations were considered statistically significant from *p* < 0.05, with *r* representing the correlation coefficient. All data are presented as mean ± standard error of the mean (SEM).

## 3. Results

### 3.1. Analysis of sMaf Expressions in the Hippocampus of AD Patients

**Figure 1:**
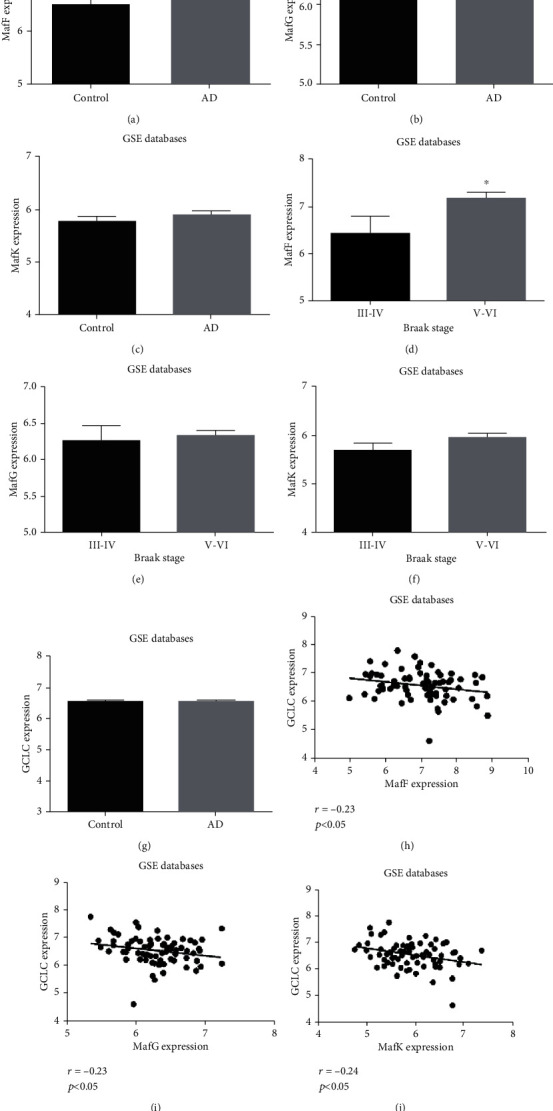
Analysis of sMaf expressions in the hippocampus of AD patients. The mRNA expressions of (a) MafF, (b) MafG, and (c) MafK in the hippocampus of AD patient databases. The mRNA expressions of (d) MafF, (e) MafG, and (f) MafK in the hippocampus of AD patients under Braak stages III-IV and V-VI. The correlations between (h) MafF, (i) MafG, and (j) MafK and GCLC in the hippocampus of AD patient databases. All data were presented as mean ± SEM. ^∗^*p* < 0.05 and ^∗∗^*p* < 0.01 versus the control group or the Braak stage III-IV group.

In order to study the expression changes of the three sMaf genes between the brains of AD patients and controls, the transcriptomic data from 74 AD patients and 66 controls from five databases were analyzed. All patients were diagnosed with AD according to stringent clinical and pathological criteria. The control group also underwent strict enrollment examinations, including cognitive and other functional tests. There was no significant difference in the mean age between the two groups. Detailed information is shown in Supplement Table S1. Compared with the control group, a significant increase in MafF expression was found in the hippocampus of AD patients (Figure 1(a)), while there were no significant changes in the expressions of MafG and MafK (Figures 1(b) and 1(c)). Furthermore, the sMaf expressions were analyzed in AD patients with definite Braak stages. We found that MafF was significantly increased in the hippocampus of patients with Braak stage V-VI, compared to those with Braak stage III-IV (Figure 1(d)). Moreover, the results of Pearson correlation analysis showed that the expressions of sMafs were negatively correlated with GCLC expression in AD patients (Figures 1(h)–1(j)). These results gave us an important reminder that MafF might participate in the pathological process of AD.

### 3.2. Constitutive Expressions of sMafs in Different Tissues

**Figure 2:**
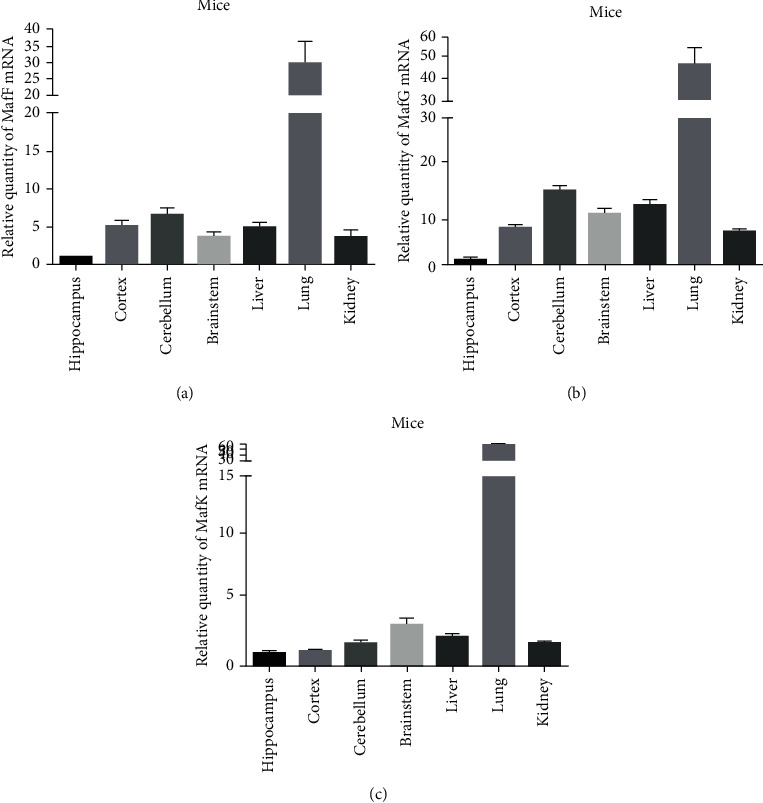
Constitutive expressions of sMafs in different tissues. The mRNA expressions of (a) MafF, (b) MafG, and (c) MafK in the brain (hippocampus, cerebral cortex, cerebellum, and brainstem), liver, lung, and kidney. The relative expressions of sMafs were normalized to GAPDH (*n* = 3). All data were presented as mean ± SEM.

Since there were less reports on the basal expressions of sMafs in tissues, especially in the brain, to document the expression of the mRNAs encoding sMafs, q-PCR was performed to determine the mRNA expressions of MafF, MafG, and MafK in different mouse brain areas, including the hippocampus, cortex, cerebellum, and brainstem, as well as from other tissues including liver, lung, and kidney. Constitutive expressions of sMafs were the highest in the lungs and relatively low in the brain (Figures 2(a)–2(c)). Basal expression levels of the three genes varied significantly in different regions of the brain. The expressions of both MafF and MafG were the highest in the cerebellum, intermediate in the cortex and the brainstem, and lowest in the hippocampus. MafF and MafG expressions were, respectively, 4-7 and 7-15 times lower in the hippocampus than in other regions of the brain (Figures 2(a) and 2(b)). MafK expression was the highest in the brainstem, intermediate in the cerebellum, and lowest in the hippocampus and the cortex. The expression of MafK in the hippocampus was similar to that found in the cortex (Figure 2(c)). Overall, the expressions of the three sMafs were lower in the brain, particularly in the hippocampus, than in other tissues.

### 3.3. MafF Expression Was Increased in response to A*β*-Induced Oxidative Injury

**Figure 3:**
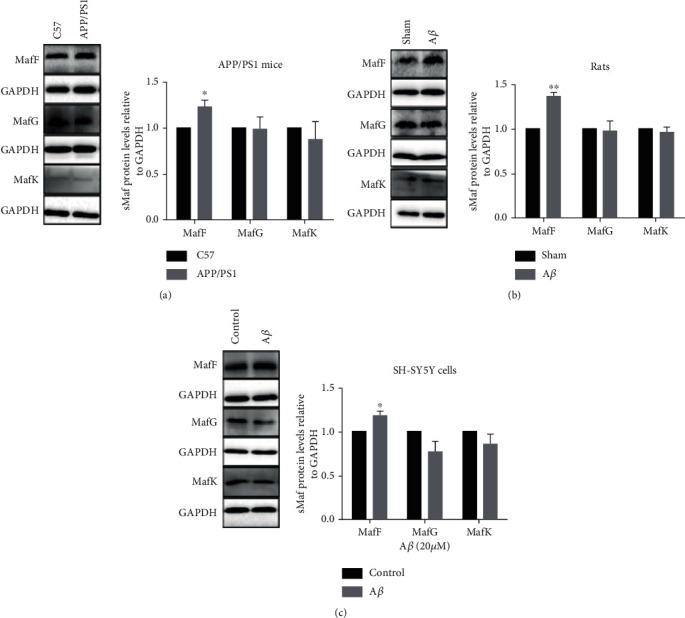
MafF expression was increased in response to A*β*-induced oxidative injury. The protein expressions of MafF, MafG, and MafK in the (a) hippocampus from APP/PS1 mice and in the (b) hippocampus from SD rats after A*β* injection and in (c) SH-SY5Y cells treated with A*β* (20 *μ*M, 48 h) (*n* = 3–5). The relative expressions of proteins were normalized to GAPDH. All data were presented as mean ± SEM; ^∗^*p* < 0.05 and ^∗∗^*p* < 0.01 versus the C57 group, the sham group, or the control group.

As A*β*-induced oxidative stress is a main cause in the development of AD, we investigated the potential changes of sMaf expressions under A*β* insult. First, 9-month-old APP/PS1 transgenic mice with numerous amyloid plaques deposited in the hippocampus were assessed to observe the expressions of sMafs under long-term A*β* insult. As shown in Figure 3(a), the MafF level was increased by 22.96% compared with that in C57 control mice, but there were no significant changes in MafG and MafK levels between the two groups of mice (Figure 3(a)). Furthermore, A*β* intrahippocampal injection rat models and A*β*-treated SH-SY5Y cells were employed to obtain the similar findings that MafF protein was upregulated by 37.63% and 19.07%, respectively (Figures 3(b) and 3(c)), and there were no significant changes in the expressions of MafG and MafK (Figures 3(b) and 3(c)).

### 3.4. MafF Might Be Involved in the Inhibition of Antioxidation Caused by A*β*-Induced Oxidative Stress

**Figure 4:**
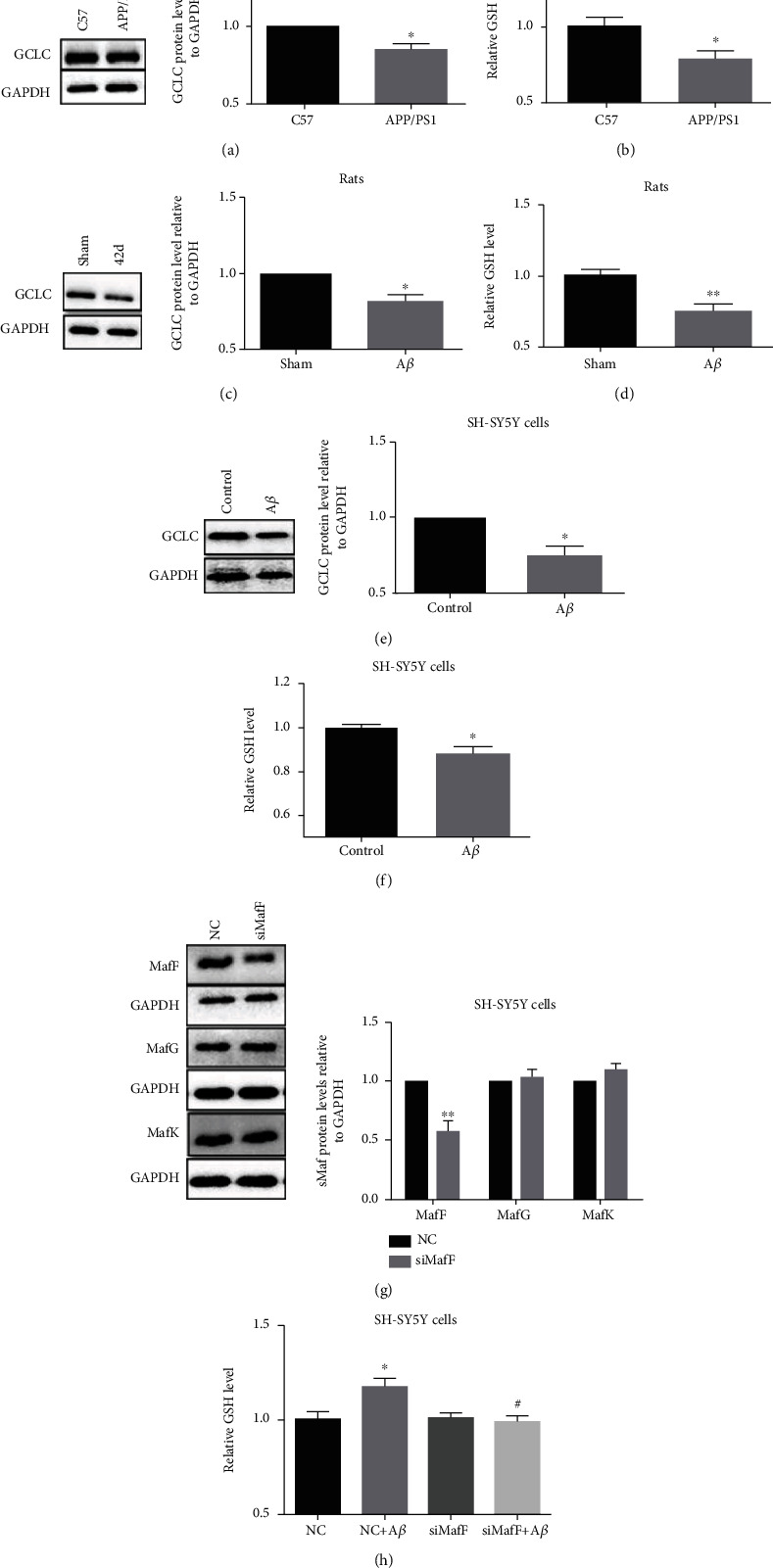
MafF expression participated in the oxidative stress caused by A*β*. The protein expressions of GCLC in the (a) hippocampus from APP/PS1 mice and in the (c) hippocampus from SD rats after A*β* injection and in (e) SH-SY5Y cells treated with A*β* (20 *μ*M, 48 h). The GSH levels in the (b) hippocampus from APP/PS1 mice and in the (d) hippocampus from SD rats after A*β* injection and in (f) SH-SY5Y cells treated with A*β* (20 *μ*M, 48 h). The protein expressions of MafF, MafG, and MafK in SH-SY5Y cells transfected with MafF siRNA for 48 h (g). GSH level in SH-SY5Y cells transfected with MafF siRNA (10 nM) for 48 h with or without A*β* (20 *μ*M) treatment (6 h) (h) (*n* = 3–7). The relative expressions of proteins were normalized to GAPDH. All data were presented as mean ± SEM; ^∗^*p* < 0.05 and ^∗∗^*p* < 0.01 versus the C57 group, the sham group, the control group, or the NC group; ^#^*p* < 0.05 versus the NC+A*β* group.

sMaf nuclear factors are important transcriptional regulators, and in particular, Nrf2/sMafs is a main endogenous pathway operating against oxidative stress [8]. This signal pathway is severely inhibited in AD patients [19]. In APP/PS1 transgenic mice, the expression of the GCLC protein was reduced by 14.89% (Figure 4(a)) and was accompanied with reduced GSH level (Figure 4(b)). In A*β* intrahippocampal injection rat models, GCLC expression was reduced by 18.21% (Figure 4(c)), and GSH was decreased by 25.56% (Figure 4(d)). Similarly, in SH-SY5Y cells treated with A*β*, GCLC was reduced by 25.59% (Figure 4(e)) and GSH level was dropped by 11.71% (Figure 4(f)).

To further investigate whether MafF was involved in GSH generation under A*β* treatment, the expression of MafF was knocked down by MafF siRNA transfection in the *in vitro* study. MafF silencing did not affect the expressions of MafG and MafK (Figure 4(g)). GSH level was increased under A*β* treatment for 6 h, but this increase was suppressed when MafF was knocked down (Figure 4(h)). The expression of MafF was closely related to the production of GSH under A*β* treatment. These results consistently suggested that MafF might be involved in the oxidative stress caused by A*β*.

### 3.5. MafF Was More Susceptible to A*β*-Induced Oxidative Stress

**Figure 5:**
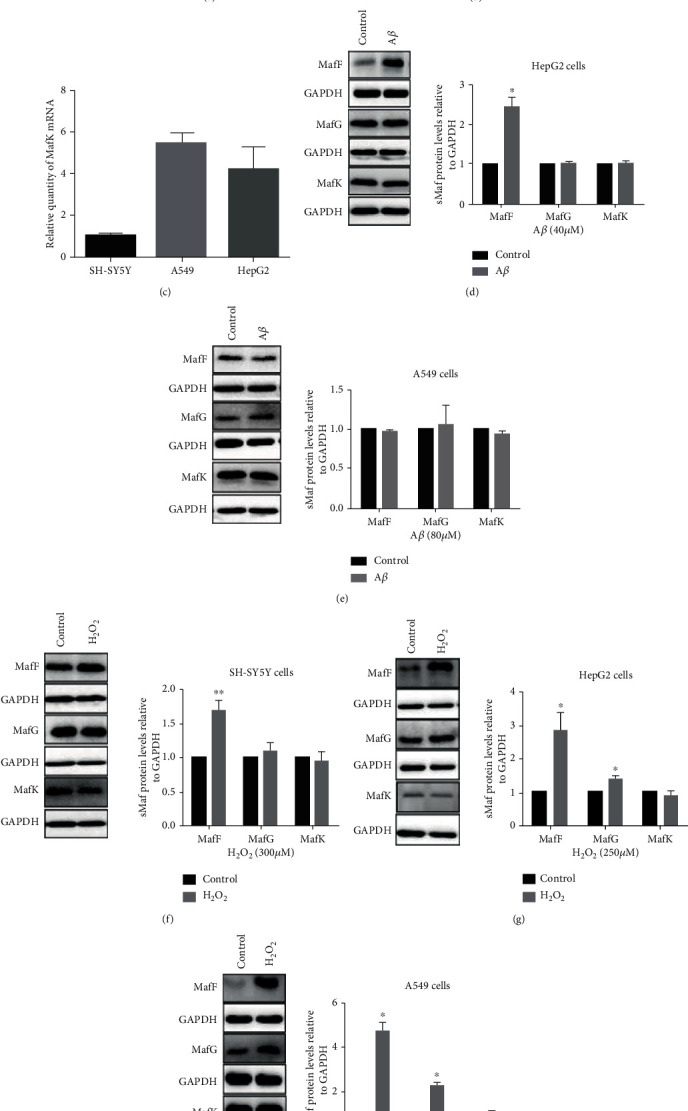
The expressions of sMafs in basal or under A*β*/H_2_O_2_ treatment in different cell lines. The constitutive expressions of (a) MafF, (b) MafG, and (c) MafK in SH-SY5Y cells, HepG2 cells, and A549 cells. The protein levels of MafF, MafG, and MafK in (f) SH-SY5Y cells, (d, g) HepG2 cells, and (e, h) A549 cells treated with A*β* for 48 h or H_2_O_2_ for 24 h (*n* = 3–5). The relative expressions of sMafs were normalized to *β*-actin/GAPDH. All data were presented as mean ± SEM; ^∗^*p* < 0.05 and ^∗∗^*p* < 0.01 versus the control group.

Furthermore, to investigate the specific effects of A*β*-induced oxidative stress on the expressions of sMafs, three different cell lines (including SH-SY5Y, HepG2, and A549) were used in the *in vitro* studies. First, we tested the constitutive expressions of MafF, MafG, and MafK mRNAs in the three cell lines. MafF, MafG, and MafK expressions were the highest in A549 cells (Figures 5(a)–5(c)). MafF expression was the lowest in SH-SY5Y cells while MafG was the lowest in HepG2 cells (Figures 5(a) and 5(b)). To determine the optimal concentration of A*β* in cell culture, we titrated the toxic effects of A*β* (Supplement Figure S1). MafF was increased by 143.99% after A*β* treatment in HepG2 cells (Figure 5(d)), whereas no significant changes were observed in the expressions of MafG and MafK. None of the protein expressions of the sMafs was significantly affected in A549 cells (Figure 5(e)). In SH-SY5Y cells, only MafF showed an increase under A*β* treatment (Figure 3(c)). These results suggested that MafF might be more susceptible to A*β*.

A strong oxidizing agent, H_2_O_2_, is usually used as a nonspecific oxidative stress inducer. We treated all three cell lines with H_2_O_2_ for 24 h. The applied concentrations of H_2_O_2_ were determined by CCK8 assay (Supplement Fig. S2). MafF was upregulated by 69.34% in SH-SY5Y cells (Figure 5(f)), while MafG and MafK remained unchanged. In HepG2 cells, MafF and MafG levels increased by 1.81-fold and 39.8%, respectively (Figure 5(g)). In A549 cells, similar to HepG2 cells, MafF and MafG levels increased by 3.75-fold and 1.31-fold, respectively, while MafK remained unchanged (Figure 5(h)). These data indicated that the expressions of sMafs are oxidative factor-specific.

## 4. Discussion

MafF, MafG, and MafK are conserved among vertebrates, including humans, mice, and rats [3]. sMaf triple-knockout mice are embryonic lethal, indicating that sMafs are indispensable during development [30]. MafF, MafG, and MafK are expressed broadly in various tissues. In this study, the tissue-specific expression profiles of the three sMaf genes were detected, which were in keeping with the reported results in mice [31]. In mouse, each sMaf gene harbors multiple first exons, which partly contribute to their tissue-specific or stimulus-specific expression patterns [3]. To our knowledge, here we for the first time reported differential expressions of sMafs in different brain regions of the mouse. The expressions of the three sMaf genes were the lowest in the hippocampus. sMafs participate in the development of diseases by affecting the level of oxidative stress. Postmortem analyses of the brain of patients with AD have documented impaired antioxidant defenses with reduction of GSH [32]. However, some postmortem studies reported elevation or no changes of GSH levels. These inconsistent results may be due to the heterogeneity of the disease stages in patients enrolled in AD [33, 34], which may also be the reason why no changes of GCLC expression in the AD patients enrolled in the studies reported in GSE databases. The growing recognition of the role of low GSH levels in AD supports the use of this characteristic as a biomarker [35]. Our present study revealed that A*β* treatment lowers the levels of GCLC and GSH, suggesting that A*β*-induced oxidative injuries played a key role in the development of AD. The results of data analysis from the AD patient databases show that all sMafs are negatively correlated with the expression of GCLC, which matches the capabilities of sMafs that they all can form homodimers to inhibit the expression of downstream GCLC [36]. However, only MafF expression is increased either in data analysis from AD patient databases or in the studies from *in vivo* and *in vitro.* Although sMafs have highly similar structures, they have distinctive functions. Torocsik et al. reported that MafK plays a crucial role in neurite outgrowth and maintenance, while MafG does not have this effect. MafF plays a crucial role in neuronal differentiation via abating epidermal growth factor- (EGF-) induced MAPK signaling [37]. In Parkinson's disease, MafF is differentially regulated in olfactory neurosphere-derived cells [38]. Our results suggest that only MafF is involved in A*β*-induced inhibition of oxidative stress. Massrieh et al. reported that MafF can be induced by interleukin-1 beta (IL-1*β*) and tumor necrosis factor (TNF) in myometrial cells, while MafG and MafK genes are not modulated by these cytokines [39]. Our earlier experiments confirmed that the levels of TNF-*α* and IL-1*β* in the hippocampus were significantly increased in A*β* intrahippocampal injection rat models, as well as in cultured cells treated with A*β*. We propose that the increases of TNF-*α* and IL-1*β* could lead to high expression of MafF under A*β* treatment, but the regulatory mechanisms of sMaf expressions need further investigation. Some peripheral biomarkers of inflammation and oxidative stress such as monomeric c-reactive protein, matrix metalloproteinases, and neutrophil-to-lymphocyte ratio have been already shown to be significantly associated with the course of disorders affecting the brain [40–43]. In the future, the expression of MafF and its relationship with peripheral biomarkers can be detected in animal models and patients at different stages of AD, and this would allow identification of a potential easy way to stage and monitor AD progression in clinical practice.

sMafs can act as transcriptional activators when they form heterodimers with Nrf2, but act as repressors when they form homodimers among themselves [36, 44, 45]. In previous studies, we found that the changes of GSH levels were varied under different time points of A*β* treatment. The level of GSH was increased at the short-term treatment of A*β* (6 h) and decreased at the long-term treatment (48 h). We speculate that the short-term treatment of A*β* increases heterodimer formation between MafF and Nrf2 and promotes the transcription of target genes, whereas the long-term treatment enhances MafF expression and promotes the formation of homodimeric repressors. GSH level increased under short-term treatment of A*β*. MafF knockdown suppressed this increase of GSH induced by A*β*, suggesting that MafF contributed to this antioxidative pathway. Moreover, MafF knockdown had no effect on the expression of MafG and MafK, which suggested that there was no complementarity between the three sMaf proteins. In addition, it has been reported that the formation of sMaf heterodimers depends on the levels of SUMOylation; it can promote the formation of sMaf heterodimers [46, 47]. Furthermore, the synthesis of GSH also depends on the supply of its substrate, such as cysteine.

According to the NCBI Reference Sequence (Ref Seq) and UCSC EST databases, human sMaf genes also have multiple alternative first exons. MafF, MafG, and MafK are expressed broadly in various tissues, but each sMaf gene has a distinctive expression profile [3]. We found that the expression patterns of the sMaf genes across different human cell lines were different. Circulating A*β* is mainly cleared by degradation in hepatocytes. Liver tissues from AD patients contain less A*β* than those from healthy individuals, which implies that the A*β*-clearance function of the liver is compromised in AD patients [48]. We observed a significant increase in MafF in HepG2 cells stimulated by A*β*. This may cause inhibition of the antioxidant capacity of liver cells. Pavliukeviciene et al. reported that A*β* oligomers inhibit the growth of A549 cells [49]. We also found that high concentrations of A*β* can significantly reduce the activity of A549 cells, but we did not observe expression changes of sMafs in A549 cell lines caused by A*β*. H_2_O_2_ is widely used as an oxidizing agent for its strong oxidation ability. A*β* can trigger the production of reactive oxygen species (ROS) as well as the accumulation of H_2_O_2_. Godoy et al. reported that quercetin completely prevents neuronal loss, ROS increase, and mitochondrial impairment in neurons exposed to H_2_O_2_. However, A*β*-induced neurotoxic effects are only partially prevented by coincubation with quercetin, which suggests that amyloid species induce neuronal damage via additional pathways [50]. Given the variability of the changes in sMaf expressions induced by A*β* and H_2_O_2_ across different cell lines, it is likely that the modulation of sMaf responses by these treatments is multifactorial. As general mechanisms, we propose that only MafF intervenes in A*β*-induced oxidative stress responses, while MafF and MafG are involved in oxidative stress responses caused by H_2_O_2_. These new hypotheses have medical implications and call for further research on the functions and regulations of sMafs. In clinical aspect, the occurrence of AD can be found by detecting the expression of MafF, and the ability of antioxidative stress can be enhanced by targeting the expression of MafF protein, thus inhibiting the further progress of AD.

## 5. Conclusion

In summary, sMafs have distinctive expression profiles in different tissues. MafF is involved in A*β*-induced oxidative stress and antioxidative pathways. The role of MafF in AD deserves further research as MafF might represent a target for AD therapy.

## Figures and Tables

**Table 1:** Primer sequences used in q-PCR.

Species	Gene	Forward primer	Reverse primer
Mice	MafF	5′-TGGGCgATGGATCTAGCCAC-3′	5′-CAACTCGCGCTTGACCTTCA-3′
	MafG	5′-GAGTGCCTGCTCACTGTGT-3′	5′-AGGTGCTGGTTCAACTCTCG-3′
	MafK	5′-GAGTCGGAACGAGAAGTCCG-3′	5′-CAGGACGGAACCACCAGAAA-3′
	GAPDH	5′-GGTTGTCTCCTGCGACTTCA-3′	5′-GGTGGTCCAGGGTTTCTTACTC-3′
Human	MafF	5′-GCCTCAGCTCCCTCCCCAAAGTG-3′	5′-ACCCCCAGGCCCAACCAGAGG-3′
	MafG	5′-AGTAAAGTCCAAGACGGATGC-3′	5′-GAAGAGAAGGAAACAGAGGGAC-3′
	MafK	5′-AGCTACGAGTTCCAGGGAG-3′	5′-ATGGACACCAGCTCATCATC-3′
	*β*-Actin	5′-GCACTCTTCCAGCCTTCC-3′	5′-TGTCCACGTCACACTTCATG-3′
	GAPDH	5′-TGGGTGTGAACCATGAGAAG-3′	5′-GAGTCCTTCCACGATACCAAAG-3′

## Data Availability

Transcriptomic data from 74 AD patients and 66 controls were collected from the databases GSE29378 (https://www.ncbi.nlm.nih.gov/geo/query/acc.cgi?acc=GSE29378), GSE36980 (https://www.ncbi.nlm.nih.gov/geo/query/acc.cgi?acc=GSE36980), GSE28146 (https://www.ncbi.nlm.nih.gov/geo/query/acc.cgi?acc=GSE28146), GSE48350 (https://www.ncbi.nlm.nih.gov/geo/query/acc.cgi?acc=GSE48350), and GSE5281 (https://www.ncbi.nlm.nih.gov/geo/query/acc.cgi?acc=GSE5281). The cross-platform normalized data were downloaded from AlzData database. The clinical information related to the samples was provided in Supplement Table S1.

## Supplementary Materials

Supplementary Materials Supplement Table S1: AD patient information in databases. Figure S1: the cell viability under A*β* treatment in different cell lines. Figure S2: the cell viability under H_2_O_2_ treatment in different cell lines.
