# Supplementary material for: Responsive Expression of MafF to β-Amyloid-Induced Oxidative Stress
Source: Dis Markers. 2020 Dec 7;2020:8861358. doi: 10.1155/2020/8861358 (PMC7787795; doi:10.1155/2020/8861358)
Supplement: Supplementary Materials — Supplement Table S1: AD patient information in databases. Figure S1: the cell viability under Aβ treatment in different cell lines. Figure S2: the cell viability under H2O2 treatment in different cell lines. [file 8861358.f1.docx]

**SupplementTable S1 AD patients information in databases**

| SampleID | Age | Disease | Sex | Braak stage | Study |
| --- | --- | --- | --- | --- | --- |
| GSM300182 | 80 | Control | male | II | GSE48350 |
| GSM300255 | 69 | Control | male | II-III | GSE48350 |
| GSM300205 | 83 | Control | male | II | GSE48350 |
| GSM300262 | 52 | Control | male | NA | GSE48350 |
| GSM300235 | 85 | Control | male | II | GSE48350 |
| GSM300197 | 74 | Control | female | 0 | GSE48350 |
| GSM300193 | 99 | Control | female | NA | GSE48350 |
| GSM300239 | 70 | Control | female | I | GSE48350 |
| GSM300190 | 74 | Control | female | II | GSE48350 |
| GSM300325 | 69 | Control | male | NA | GSE48350 |
| GSM300243 | 64 | Control | female | NA | GSE48350 |
| GSM300268 | 86 | Control | male | I-II | GSE48350 |
| GSM300223 | 74 | Control | female | II | GSE48350 |
| GSM300286 | 69 | Control | male | I | GSE48350 |
| GSM300178 | 95 | Control | male | I-II | GSE48350 |
| GSM300339 | 82 | Control | female | II | GSE48350 |
| GSM300215 | 97 | Control | male | II | GSE48350 |
| GSM300333 | 75 | Control | male | NA | GSE48350 |
| GSM300329 | 91 | Control | female | II | GSE48350 |
| GSM119628 | 85 | Control | male | I-II | GSE5281 |
| GSM119629 | 80 | Control | male | I-II | GSE5281 |
| GSM119630 | 80 | Control | male | I-II | GSE5281 |
| GSM119631 | 102 | Control | female | I-II | GSE5281 |
| GSM119632 | 63 | Control | male | I-II | GSE5281 |
| GSM119633 | 79 | Control | male | I-II | GSE5281 |
| GSM119634 | 76 | Control | male | I-II | GSE5281 |
| GSM119635 | 83 | Control | male | I-II | GSE5281 |
| GSM119636 | 79 | Control | male | I-II | GSE5281 |
| GSM119637 | 88 | Control | female | I-II | GSE5281 |
| GSM119638 | 73 | Control | female | I-II | GSE5281 |
| GSM119639 | 69 | Control | male | I-II | GSE5281 |
| GSM119640 | 78 | Control | male | I-II | GSE5281 |
| GSM726086 | 86 | Control | male | I | GSE29378 |
| GSM726102 | 73 | Control | male | II | GSE29378 |
| GSM726089 | 88 | Control | female | NA | GSE29378 |
| GSM726109 | 85 | Control | male | II | GSE29378 |
| GSM726097 | 90 | Control | male | II | GSE29378 |
| GSM726130 | 80 | Control | male | NA | GSE29378 |
| GSM726139 | 90 | Control | male | I | GSE29378 |
| GSM726124 | 75 | Control | male | I | GSE29378 |
| GSM726085 | 72 | Control | female | NA | GSE29378 |
| GSM726100 | 83 | Control | female | I | GSE29378 |
| GSM726099 | 74 | Control | female | II | GSE29378 |
| GSM726116 | 70 | Control | female | I | GSE29378 |
| GSM726105 | 85 | Control | male | II | GSE29378 |
| GSM726121 | 84 | Control | male | NA | GSE29378 |
| GSM726134 | 81 | Control | male | II | GSE29378 |
| GSM726117 | 90 | Control | male | I | GSE29378 |
| GSM907865 | 55 | Control | male | I | GSE36980 |
| GSM907869 | 80 | Control | male | II | GSE36980 |
| GSM907862 | 80 | Control | female | I | GSE36980 |
| GSM907867 | 78 | Control | female | I | GSE36980 |
| GSM907863 | 84 | Control | female | IV | GSE36980 |
| GSM907864 | 77 | Control | male | NA | GSE36980 |
| GSM907870 | 74 | Control | male | NA | GSE36980 |
| GSM907868 | 83 | Control | male | IV | GSE36980 |
| GSM907861 | 87 | Control | female | III | GSE36980 |
| GSM907866 | 72 | Control | female | I | GSE36980 |
| GSM697308 | 85 | Control | male | - | GSE28146 |
| GSM697309 | 80 | Control | male | - | GSE28146 |
| GSM697310 | 92 | Control | female | - | GSE28146 |
| GSM697311 | 80 | Control | male | - | GSE28146 |
| GSM697312 | 75 | Control | male | - | GSE28146 |
| GSM697313 | 97 | Control | female | - | GSE28146 |
| GSM697314 | 95 | Control | male | - | GSE28146 |
| GSM697315 | 87 | Control | male | - | GSE28146 |
| GSM1176212 | 74 | AD | female | VI | GSE48350 |
| GSM1176220 | 91 | AD | female | V-VI | GSE48350 |
| GSM1176213 | 76 | AD | female | VI | GSE48350 |
| GSM1176226 | 86 | AD | male | IV | GSE48350 |
| GSM1176223 | 79 | AD | male | VI | GSE48350 |
| GSM1176225 | 85 | AD | male | III | GSE48350 |
| GSM1176224 | 80 | AD | male | NA | GSE48350 |
| GSM1176211 | 60 | AD | female | NA | GSE48350 |
| GSM1176227 | 87 | AD | male | VI | GSE48350 |
| GSM1176228 | 94 | AD | male | VI | GSE48350 |
| GSM1176222 | 76 | AD | male | III | GSE48350 |
| GSM1176216 | 85 | AD | female | IV | GSE48350 |
| GSM1176221 | 76 | AD | male | VI | GSE48350 |
| GSM1176219 | 90 | AD | female | III | GSE48350 |
| GSM1176214 | 79 | AD | female | VI | GSE48350 |
| GSM1176218 | 90 | AD | female | IV | GSE48350 |
| GSM1176215 | 86 | AD | female | NA | GSE48350 |
| GSM1176229 | 94 | AD | male | IV | GSE48350 |
| GSM1176217 | 90 | AD | female | VI | GSE48350 |
| GSM238799 | 95 | AD | female | V-VI | GSE5281 |
| GSM238800 | 68 | AD | male | V-VI | GSE5281 |
| GSM238801 | 95 | AD | female | V-VI | GSE5281 |
| GSM238802 | 70.8 | AD | female | V-VI | GSE5281 |
| GSM238803 | 85 | AD | female | V-VI | GSE5281 |
| GSM238804 | 83 | AD | female | V-VI | GSE5281 |
| GSM238805 | 77 | AD | female | V-VI | GSE5281 |
| GSM238806 | 83 | AD | female | V-VI | GSE5281 |
| GSM238807 | 68 | AD | male | V-VI | GSE5281 |
| GSM238808 | 79 | AD | male | V-VI | GSE5281 |
| GSM726128 | 90 | AD | male | V | GSE29378 |
| GSM726111 | 84 | AD | female | VI | GSE29378 |
| GSM726145 | 68 | AD | female | V | GSE29378 |
| GSM726101 | 83 | AD | male | VI | GSE29378 |
| GSM726093 | 66 | AD | male | NA | GSE29378 |
| GSM726138 | 72 | AD | male | V | GSE29378 |
| GSM726094 | 67 | AD | male | VI | GSE29378 |
| GSM726127 | 79 | AD | female | VI | GSE29378 |
| GSM726091 | 61 | AD | female | NA | GSE29378 |
| GSM726129 | 74 | AD | female | IV | GSE29378 |
| GSM726087 | 81 | AD | male | V | GSE29378 |
| GSM726118 | 68 | AD | male | V | GSE29378 |
| GSM726108 | 80 | AD | female | V | GSE29378 |
| GSM726115 | 81 | AD | male | V | GSE29378 |
| GSM726107 | 90 | AD | female | VI | GSE29378 |
| GSM726120 | 79 | AD | female | V | GSE29378 |
| GSM907859 | 83 | AD | male | VI | GSE36980 |
| GSM907854 | 88 | AD | female | V | GSE36980 |
| GSM907858 | 99 | AD | male | V | GSE36980 |
| GSM907855 | 95 | AD | female | VI | GSE36980 |
| GSM907860 | 90 | AD | male | V | GSE36980 |
| GSM907856 | 95 | AD | female | VI | GSE36980 |
| GSM907857 | 100 | AD | female | VI | GSE36980 |
| GSM697318 | 83 | AD | female | - | GSE28146 |
| GSM697319 | 88 | AD | male | - | GSE28146 |
| GSM697320 | 91 | AD | female | - | GSE28146 |
| GSM697321 | 88 | AD | male | - | GSE28146 |
| GSM697322 | 97 | AD | female | - | GSE28146 |
| GSM697323 | 85 | AD | female | - | GSE28146 |
| GSM697324 | 89 | AD | female | - | GSE28146 |
| GSM697325 | 83 | AD | female | - | GSE28146 |
| GSM697326 | 82 | AD | female | - | GSE28146 |
| GSM697327 | 79 | AD | female | - | GSE28146 |
| GSM697328 | 81 | AD | male | - | GSE28146 |
| GSM697329 | 86 | AD | female | - | GSE28146 |
| GSM697330 | 82 | AD | male | - | GSE28146 |
| GSM697331 | 85 | AD | male | - | GSE28146 |
| GSM697332 | 65 | AD | female | - | GSE28146 |
| GSM697333 | 93 | AD | male | - | GSE28146 |
| GSM697334 | 79 | AD | female | - | GSE28146 |
| GSM697335 | 94 | AD | female | - | GSE28146 |
| GSM697336 | 79 | AD | female | - | GSE28146 |
| GSM697337 | 93 | AD | female | - | GSE28146 |
| GSM697316 | 101 | AD | female | - | GSE28146 |
| GSM697317 | 95 | AD | female | - | GSE28146 |

**
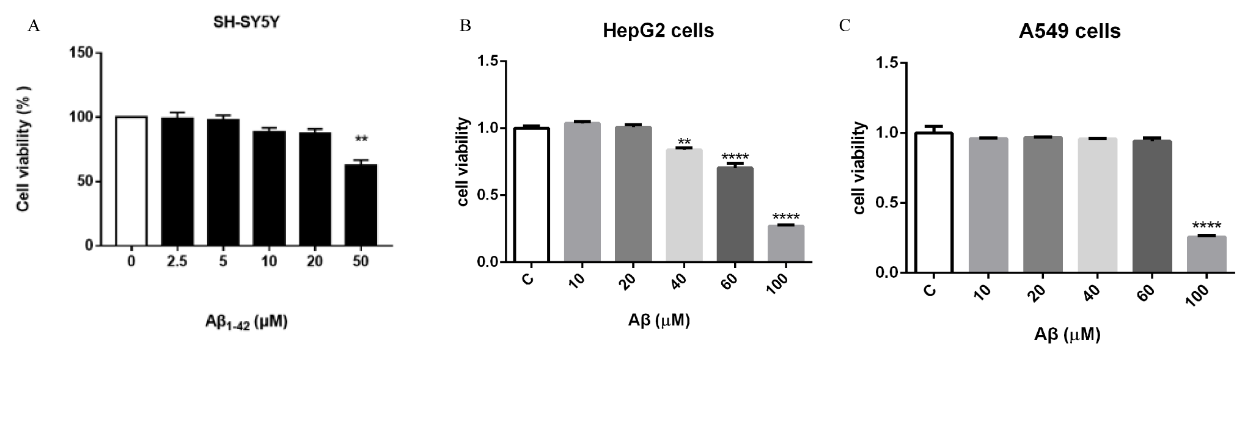
**

**Figure S1 The cell viability under Aβ treatment in different cell lines.**

MTT assay was used to assess the effects of Aβ on SH-SY5Y cells (n=5) . CCK8 assay was used to assess the effects of Aβ on HepG2 and A 549 cells (n=3-5) . All data were presented as mean ± SEM. ***p* < 0.01 versus control group, *****p* < 0.0001 versus control group.


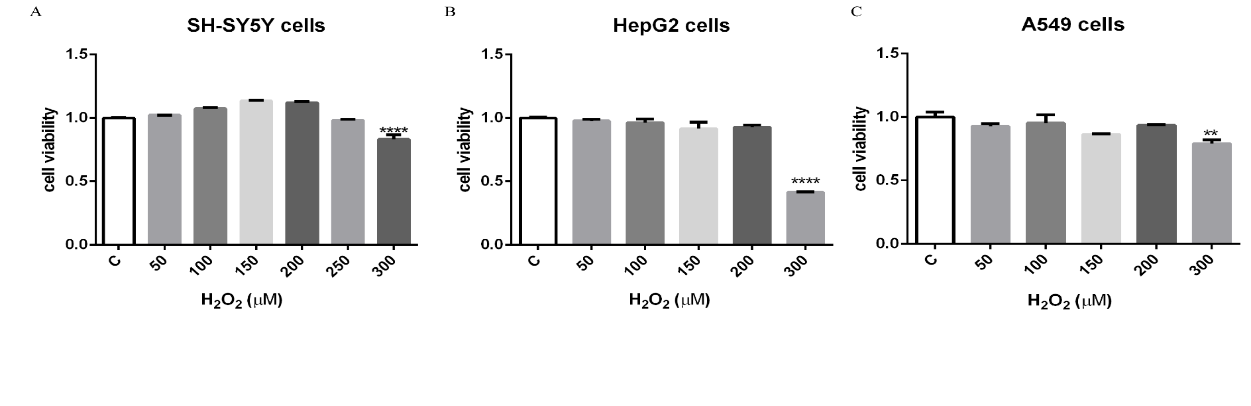
 **Figure S2 The cell viability under H_2_O_2_ treatment in different cell lines.**

CCK8 assay was used to assess the effects of H_2_O_2_ on SH-SY5Y, HepG2 and A 549 cells (n=3-4). All data were presented as mean ± SEM. ***p* < 0.01 versus control group, *****p* < 0.0001 versus control group.
